# Sub-microscopic gametocyte carriage in febrile children living in different areas of Gabon

**DOI:** 10.1186/1475-2875-12-375

**Published:** 2013-10-29

**Authors:** Denise Patricia Mawili-Mboumba, Rosalie Nikiéma, Marielle Karine Bouyou-Akotet, Noemi Bahamontes-Rosa, Alfred Traoré, Maryvonne Kombila

**Affiliations:** 1 Department of Parasitology-Mycology, Faculty of Medicine, Université des Sciences de la Santé, BP 4009, Libreville, Gabon; 2 Diseases of the Developing World, GlaxoSmithKline, 28760 Tres Cantos, Madrid, Spain; 3 Department of Biochemistry-Microbiology, UFR Sciences de la Vie et de la Terre, University of Ouagadougou, 03 BP 7012 03, Ouagadougou, Burkina Faso

**Keywords:** *Plasmodium falciparum*, Submicroscopic gametocytes, Transmission, Children, Gabon

## Abstract

**Background:**

Considering malaria prevalence declines in parts of sub-Saharan Africa, such as Gabon, identification of the human infectious reservoir is important for successful malaria control. Microscopic and sub-microscopic parasites contribute to malaria transmission. The aim of the present study was to evaluate the proportion of microscopic and sub-microscopic gametocyte carriers among febrile patients in two different areas of Gabon.

**Methods:**

Samples from febrile children aged less than 11 years old were collected from February 2008 to January 2009 at two health centres of Gabon. Patients were screened for the presence of asexual *Plasmodium falciparum* parasites. Gametocyte carriage was determined by microscopy and QT-NASBA.

**Results:**

Gametocytes were detected in 5.3% (n = 16/304) of children by microscopy compared to 45.7% (n = 139/304) by QT-Nasba. Sub-microscopic gametocyte carriage (ie microscopy negative and QT-Nasba positive) was found in 89.2% (n = 124/139) of patients. Among patients with microscopically detected trophozoites, the proportion of sub-microscopic gametocyte (SMG) carriers was 58.4% (n = 118/202) and 6% in samples from children with negative slides (*p* < 0.01). In Oyem, where malaria prevalence is three-fold higher than in Owendo, SMG carriage was more frequent (49.0% *vs* 32.6% in Owendo; *p* < 0.01).

**Conclusion:**

Sub-microscopic gametocytaemia is common among Gabonese febrile children. They might strongly contribute to maintain malaria transmission. However, further analysis of sub-microscopic parasite carriage among asymptomatic individuals will be helpful to better characterize malaria transmission.

## Background

A trend towards a decrease of malaria prevalence was noticed in many malaria endemic areas of sub-Saharan Africa [1-5]. To maintain malaria control and elimination efforts, targeting gametocytes, either alone or as part of integrated control programmes, is essential [6,7]. Indeed, microscopic and sub-microscopic gametocytes sustain malaria transmission. Their presence in human peripheral blood and their infectiousness determine the success of transmission from human to mosquitoes [8-10]. It is assumed that a little proportion of patients with a primary infection develop patent gametocytaemia, a good indicator to characterize the level of transmission in endemic areas [11]. However, sexual and asexual sub-microscopic infection carriers are frequent and constitute an important parasite reservoir, mainly among asymptomatic and target population even after treatment [11-13]. They are barriers to interrupt malaria transmission and to the successful of malaria elimination. Therefore, the importance of the prevalence of gametocyte carriers as human infectious reservoir needs to be investigated. A good anti-malarial drug must have a high gametocytocidal activity. However recent data suggest that submicroscopic gametocytes carriage could persist after anti-malarial treatment [14,15]. This duration of gametocytaemia is increased in case of drug resistant parasite carriage and maintains the spread of drug resistance [16,17]. Therefore, the presence of submicroscopic gametocytes following anti-malarial treatment could be a possible marker of resistant parasites, and a valuable indicator of drug efficacy, that could be assessed using molecular methods.

Compared to microscopy, molecular techniques were developed and applied with good results for *Plasmodium falciparum* asexual and sexual forms detection [18,19]. The Quantitative Nucleic Acid Based-Amplification (QT-NASBA) has been reported to be a highly sensitive molecular method for sub-microscopic gametocytes detection and quantification [18]. The use of QT-Nasba revealed that individuals living in malaria endemic areas may carry sub-microscopic gametocytes that can lead to *Anopheles* infection [20,21].

In Gabon, a change was observed in malaria epidemiology and in the profile of the target population. A decrease of malaria prevalence was observed between 2005 and 2008 followed by a rebound in 2011 [22,23]. Nevertheless, few data on malaria transmission are available since the implementation of artemisinin-based combination therapy (ACT) in Gabon. Likewise, data on gametocyte carriage are scarce. In the present study, the proportion of microscopic and sub-microscopic gametocytes (SMG) carriers was evaluated among febrile patients in two areas of Gabon with different level of malaria prevalence.

## Methods

### Study areas

The study was conducted in Gabon where malaria transmission is perennial and predominantly due to *P*. *falciparum*. From February 2008 to January 2009, recruitment was carried out at the Centre Hospitalier Régional d’Oyem (CHRO) and the Centre de Santé Communautaire d’Owendo (CSCO), 25 km south of Libreville, the capital city of Gabon [24]. Malaria prevalence among febrile children was 39% and 11% in Oyem and Owendo, respectively.

### Sample collection

This study was a part of a health facility-based project initiated by the National Malaria Control Programme (NMCP), which aimed to evaluate malaria prevalence and to perform molecular analysis of *P*. *falciparum* isolates. Children under 11 years of age, presenting at hospital for a consultation with fever (axillary temperature ≥ 37.5°C) or a history of fever in the preceding 48 h, were enrolled from February 2008 to January 2009. Body temperature, history of fever and sex were collected from each patient. During this prospective study that lasted one year, a single blood sample was taken from each febrile patient included, for malaria diagnosis. Fifty microlitres of blood were spread on filter papers for molecular testing that include analysis of genetic diversity, drug resistance markers and SMG detection. Filter papers were dried and stored in individual plastic bags at room temperature (20-25°C) until processed. A random subset of approximately 16 samples with microscopic *P*. *falciparum* infection and eight negative samples were selected per month by personal blinded to all information concerning the patients and the microscopic results.

### Malaria diagnosis

Malaria diagnosis was performed according to the method of Lambarene, as detailed elsewhere [25]. Briefly, 10 μL of blood is distributed on a 10- by 18-mm area of a microscope slide, which is dried and stained by a 20% Giemsa solution. Experienced microscopists read the smears using a light microscope with the 100× objective under oil immersion (×1,000 magnification). Parasitaemia was expressed as a number of parasites per microlitre of blood. Parasites stages (microscopic asexual forms and gametocytes) density was determined. Parasite species was identified in the matched thin blood smears. Smears were considered as negative if the examination of 100 oil immersion fields did not reveal any parasites. They were classified as positive in case of a positive blood smear (PBS), irrespective of the parasite density, and as negative when microscopic examination was negative. Patients with a positive blood smears with a non-*falciparum* species were not selected.

### Gametocyte detection: nucleic acid extraction and *Pfs25* mRNA real-time QT-NASBA

Nucleic acids were extracted from blood collected on filter paper with the guanidiumisothiocyanate (GuSCN)/silica procedure using semi-automated analyzer Mini-Mag (Biomérieux®). *Pfs25* mRNA QT-NASBA was performed as previously described [8]. Briefly, real-time QT-NASBA for *Pfs25* mRNA was done on a Nuclisens EasyQ analyser (Biomérieux®, France). The Nuclisens Basic Kit served for amplification according to the manufacturer’s instructions at a KCl concentration of 80 mmol/L with specific primers. For *Pfs25* mRNA, the forward primer was: 5’-gac tgt aaa taa acc atg tgg aga-3’; the reverse primer was: 5’-aat tct aat acg act cac tat agg gag aag gca ttt acc gtt acc aca agt ta-3’. *Pfs25* molecular beacon was 5’-TexasRed-cga tcg ccg ttt cat acg ctt gta acg atc g-DABSYL-3’.

Each reaction was performed in a total volume of 10 μL. For gametocyte detection, a culture of 3D7 *P*. *falciparum* mature gametocytes, with a parasitaemia of 0.35%, was used in duplicate during each experiment as positive control. Samples were also analysed in duplicate and considered positive when the time-point of amplification at which the fluorescence detecting target amplicons (time to positivity = TTP) exceeded the mean fluorescence of three negative controls plus 20 standard deviation (SD).

### Ethical aspects

This study was a part of a project designed by the MNCP for morbidity and diagnostic tools evaluation in Gabon. Molecular analysis for gametocyte detection and drug resistance was part of this project. Gabonese Ministry of Health approved the entire study. Febrile children's parents or guardians were informed about the study protocol, either for the comparison of diagnostic tools or for the consecutive molecular analysis. Their oral consent was required prior to enrolment.

### Sample size estimation

Sample size was calculated by considering i) the previous data estimating that 10% of febrile children have microscopic gametocytaemia [26]; ii) the prevalence of gametocytes detected by *PfS25* being at least twice of that detected by microscopy in clinic patients [11], iii) at least 60% of ill patients with only microscopic asexual forms can have sub-microscopic gametocytaemia [9,27]. Sample size calculation was done using STATA software and Epi-Info 6 with a risk of 5% and accuracy of 10%. Therefore, a minimum number of 60 samples per site was estimated. However, considering that in perennial transmission areas, 10 to 30% of febrile children have sub-microscopic *P*. *falciparum* infections additional microscopically negative samples were collected (30%). A minimum of 60 dried blood spot from patients with positive blood smears and 30 samples from individuals microscopically negative was then required.

### Data analysis

Data were entered using Epi info version 2000 and were analysed with Stata 9.2 (Stata Corporation, College Station, TX USA). SMG carriers were defined as indiduals harbouring gametocytes exclusively detected with Nasba. Continuous variables are presented as medians [25^th^ and 75^th^ percentiles]. Differences between groups were assessed using chi-squared or Fisher’s exact tests for proportions. Continuous variables were compared using the student t-test, Mann Whitney U-test or Kruskal-Wallis test as appropriate. Spearman’s test was used to assess the correlation between continuous variables. A *p*-value of less than 0.05 was considered significant.

## Results

Samples from 304 children were analysed. Half of them (n = 153) were collected from patients living in Owendo. The median age of the children was 36 [21-27] months; those from Oyem were the youngest (Table 1); 54.9% were male.

**Table 1:** Characteristics of the study population

	**All**	**Oyem (CHRO)**	**Owendo (CSCO)**	p-value*
	**n = 304**	**n = 151**	**n = 153**	
Age, median [IQR], months	36[21-72]	24[16-48]	48[24-84]	< 0.01
T°, median [IQR], C	38[37.1-39.1	38.5[37.5-39.5]	37.7[36.9-38.7]	< 0.01
Malaria infection				
*Microscopy*				
MPBS** (trophozoïtes), n (%)	202 (66.4)	102 (67.5)	100 (65.3)	0.9
Trophozoïtes density, median [IQR] P/μL	26110[3024-111860]	34480[3854-122675]	18008[2742-96600]	0.2
MPBS* (gametocytes) n (%)	16 (5.3)	5 (3.3)	11 (7.2)	0.12
Gametocytes density, median [IQR] P/μL	119[23-875]	119[30-423]	1053[7-2100]	0.9
*Nasba*				
Gametocytes n (%)	139 (45.7)	79 (52.3)	60 (39.2)	0.02
SMG*** n (%)	124 (40.7)	74 (49.0)	50 (32.6)	< 0.01

*for comparison between Oyem and Owendo; MPBS**: malaria positive blood smears; SMG***: sub-microscopic gametocytes.

### Microscopic *P*. *falciparum* sexual and asexual forms

A total of 202 (66.4%) samples from patients with positive blood smears were alternatively collected in the two sites (p = 0.9). The median parasite density was 26,110 IQR [3,024-111,860] trophozoites/μl (Table 1). Gametocytes were detected in 5.3% (n = 16) of sample samples with asexual forms only. The median gametocytaemia was of 119 IQR[23-875] gametocytes/μL. More than half of the 16 microscopic gametocyte carriers (n = 11; 68.7%) lived in Owendo (Table 1).

### *Plasmodium falciparum* sub-microscopic gametocytes carriers

Based on molecular analysis, 139 (45.7%) samples carried gametocytes, of which 89.2% (n = 124/139) were sub-microscopic. The presence of mature gametocytes was confirmed by *Pfs25 mRNA QT*-*NASBA* in 15 of the 16 samples with gametocytes detected on blood smears. The microscopic parasite density was greater than 100 gametocytes/μl, in 14 out of the 15 samples (Table 2). Molecular detection was not successful in one sample with 7 gametocytes/μl detected by microscopy.

**Table 2:** Proportion of gametocytes detected by Nasba according to the presence of microscopic trophozoïtes and gametocytes

	**Gametocytes detection (Nasba)**		
	**Detected**	**Not detected**	**Total**
Microscopy			
Trophozoites			
-Positive slides, n (%)	133 (65.8)	69 (34.2)	202
-Negative slides, n (%)	6 (5.9)	96 (94.1)	102
Gametocytes			
-Positive slides, n (%)	15 (93.7)	1 (6.3)	16
-Density (gametocytes/μl)			
0	118 (40.9)	170 (59.1)	288
1-50	1 (50.0)	1 (50.0)	2
51-500	5 (100.0)	0 (0.0)	5
501-1000	5 (100.0)	0 (0.0)	5
> 1000	4 (100.0)	0 (0.0)	4

The proportion of *Pfs25* QT-Nasba gametocyte carriers was higher in Oyem (52.3%) compared to Owendo (39.2%) (p < 0.02). A similar trend was observed for sub-microscopic gametocytes carriage (ie negative blood smears with positive QT-Nasba amplification) that was significantly more frequent in Oyem (49.0%; n = 74/151 *vs* 32.7%; 50/153 in Owendo) (Table 1). The proportion of gametocytes only detected by QT-Nasba was 58.4% (n = 118) among patients with microscopically detected trophozoites and 6% among those with negative slides (*p* < 0.01).

## Discussion

Data on gametocyte carriage in population from malaria endemic areas are needed to estimate the infectious reservoir and battle the ongoing transmission of malaria. This is important in the light of renewed malaria elimination and eradication efforts [28].

QT-NASBA gametocytes detection is currently one of the best methods to detect sub-microscopic gametocytes. It is usually performed with mRNA extracted from frozen blood sample pellets. Few authors reported gametocytes detection by using filter paper with Nasba technology [29-31]. Recently, RNA extraction from infected red blood cells produced in culture, spotted on filter paper was done and its analysis using QT-Nasba led to good results [30,31]. In the present study, nucleic acids were extracted from field *P*. *falciparum* isolates collected on filter paper and stored at room temperature (20-25°C). All, but one, samples detected positives by microscopy were also positive with QT-NASBA. Therefore, RNA extracts analysis from non-frozen blood samples stored under stable conditions give reliable results for determining gametocyte prevalence. The lack of amplification in one sample could be due to several factors, such as the gametocyte density (< 10/μl) or an unsuccessful RNA extraction although a semi-automated analyzer was used. Inclusion of a control to assess the extraction efficiency would have been informative, but this is not currently performed as reported in previous studies [8,20,32]. Nevertheless, the suitability of this approach in areas without modern equipment has been confirmed with culture-produced gametocytes, as well as field isolates collected on filter paper [30,31].

In the present study, 5.3% of the febrile children screened had microscopically detected gametocytes. Using molecular detection, this proportion increased to 45.7%, whose 90% were sub-microscopic gametocytes carriers. The higher rate of gametocytes detected by QT-Nasba compared to microscopy (ranging from 15% to 91%) has been frequently reported by other authors [9,32-34].

The results obtained here confirm the high frequency of sub-microscopic gametocytes carriage among symptomatic patients harbouring microscopically detected asexual *P*. *falciparum* forms (58.4%) as reported elsewhere [35]. This proportion can reach 90% [33,34].

According to the study site, the profile of sub-microscopic gametocytes carriage is the same as the microscopic *P*. *falciparum* asexual form prevalence. Both, sexual and asexual *P*. *falciparum* infections, are more frequently observed in children living in Oyem (52.3% and 39%) compared to Owendo (39.2% and 11%) [24]. Okell *et al* showed a relationship between malaria prevalence by microscopy and PCR when they analysed 106 surveys [13]. A survey performed three years later in Oyem and in Libreville, located at 25Kms from Owendo, confirmed the higher burden of malaria in the area of Oyem [23]. It will be interesting to study the sub-microscopic malaria prevalence in patients living in areas with a lower endemicity, such as Owendo. Indeed, a higher frequency of sub-microscopic infection is often described in settings where slide prevalence is below 24% [13].

All sub-microscopic gametocytes found in the present study were detected in blood samples collected from febrile children before any medical prescription. This finding is important in the current context of malaria strategies control update in Gabon where a rebound of malaria prevalence is observed [23]. In fact, most of the anti-malarial drugs have a limited activity against late stage of *P*. *falciparum* gametocytes [14,35]. Although ACT shortens the duration of post-treatment circulation of mature gametocytes, a low density of mature sexual forms can persist after apparently complete clearance of trophozoites [14,35]. On day 28 after ACT treatment, a proportion of 12-40% of cured patients was found to be still gametocytaemic; a status which allows post-treatment malaria transmission [9,14,36,37]. Therefore, it can be assumed that the rebound of *P*. *falciparum* infections in Gabon between 2005 and 2008 could be partly explained by the importance of the sub-microscopic infectious reservoir (above 40%) that maintains a continuous transmission. Microscopic or sub-microscopic gametocyte carriage contributes to *Anopheles* infection and is responsible for transmission sustainment [20]. Taken altogether, these observations highlight the need of a treatment with gametocytocidal drugs. Adding primaquine (PQ) to ACT is suggested to enhance anti-malarial drugs impact on malaria transmission, although concerns about PQ treatment induced haemolysis in case of G6PD deficiency exist [38,39].

This study presents some limits. sub-microscopic gametocytes carriage was only determined in symptomatic children. Thus, it is probably underestimated when considering that asymptomatic microscopic and sub-microscopic infections carriers are frequent in high but also low transmission settings whatever the seasonality [13,40]. The sample selection did not allow an analysis of sub-microscopic gametocytes prevalence according to the age. Gametocyte viability and infectivity to mosquito were not evaluated. It is however already known that sub-microscopic gametocytes are responsible for 20-50% of transmission to mosquito [13,20].

## Conclusion

Frequent sub-microscopic gametocytaemia among children presenting at clinics is observed in areas of Gabon with different malaria burden. They would constitute an important reservoir of parasites, even in Owendo where malaria prevalence tends to be low. Therefore, further studies analysing the sub-microscopic *P*. *falciparum* prevalence including gametocytes carriage in asymptomatic children and adults, the acquired immunity in different malaria intensity areas and entomological data will be helpful to highlight the characterization of the disease transmission in the country.

## Competing interests

The authors declare that they have no competing interests.

## Authors’ contributions

The study was initiated by MK and AT. DPMM and RN conducted the study. DPMM, RN and MKBA interpreted the data and drafted the manuscript. Data were analysed by MKBA. NBR did the parasite culture. All authors revised and approved the final manuscript.
